# Changes in training activity post COVID-19 infection in recreational runners and cyclists

**DOI:** 10.17159/2078-516X/2022/v34i1a13758

**Published:** 2022-01-01

**Authors:** A Emeran, EV Lambert, T Paruk, A Bosch

**Affiliations:** 1 UCT Research Centre for Health through Physical Activity Lifestyle and Sport (HPALS), Department of Human Biology, Faculty of Health Sciences, University of Cape Town, Cape Town, South Africa; 2 International Federation of Sports Medicine (FIMS) Collaborative Centre of Sports Medicine, University of Cape Town, Cape Town, South Africa; 3 National Research Foundation (NRF), Cape Town, South Africa

**Keywords:** SARS-CoV-2, coronavirus, physical activity, training activity, relative exercise intensity

## Abstract

**Background:**

Anecdotal evidence suggests that athletes struggle to return to exercise post COVID-19 infection. However, studies evaluating the effect of COVID-19 on athletes’ exercise activity are limited.

**Objectives:**

The objectives of this study were: (i) to describe the perceptions of recreational runners and cyclists recovering from COVID-19 on their training activity and general well-being, (ii) to compare device-measured training data in runners and cyclists pre- and post COVID-19, with non-infected controls that had a training interruption.

**Methods:**

Participants who were recruited via social media completed an online questionnaire (n=61), including demographic, health and COVID-19 descriptive data. In a sub-sample, device-measured training data (heart rate, time, distance and speed, n=27) were obtained from GPS devices for four weeks before infection and on resumption of training. Similar data were collected for the control group (n=9) whose training had been interrupted but by factors excluding COVID-19.

**Results:**

Most participants experienced a mild to moderate illness (91%) that was associated with a training interruption time of two-four weeks. Decreases in heart rate, relative exercise intensity, speed, time and distance were observed during the first week of returning to training for both groups, followed by an increase from Week two onwards.

**Discussion:**

Results failed to support a ‘COVID-19 effect’ on exercise activity as reductions in training variables occurred in both the COVID-19 and control groups. A possible explanation for the reductions observed is a deliberate gradual return to training by athletes post-COVID-19.

**Conclusion:**

More research is needed using device-measured training data prior to and post COVID-19 infection to better understand the impact of the SARS-CoV-2 virus on the exercise activity of athletes.

The COVID-19 pandemic has had a major impact on morbidity and mortality globally.^[1]^ Fortunately, most COVID-19 cases have been classified as mild to moderate.^[2]^ However, even with mild illness severity, individuals may still require a prolonged time to full recovery, particularly when returning to exercise. Anecdotal evidence shows that many individuals, including athletes, have difficulty returning to their normal level of exercise post infection.^[3]^ Symptoms challenging athletes’ return to exercise include coughing, tachycardia, and fatigue.^[4]^ There is limited research on the effect of COVID-19 on the exercise activity of athletes post infection, with several studies reporting minimal impairment of exercise capacity on cardiopulmonary exercise testing (CPET) in athletes post COVID-19.^[5,6]^ To our knowledge, no research has been undertaken on objectively measured training data obtained from runners and cyclists pre- and post COVID-19 infection.

The current study aims to address the gap in knowledge for the above by describing the perceptions of recreational runners and cyclists recovering from COVID-19 on their training activity and general well-being. In a sub-sample, we will compare objectively measured training data in runners and cyclists pre- and post COVID-19 infection with non-infected controls who experienced a training interruption. The study differs from existing studies, as instead of using exercise testing, exercise data from participants’ Global Positioning System (GPS) wearable devices were used to determine whether a change in exercise activity occurred post infection. To our knowledge, this is the first study to measure exercise activity of athletes pre- and post infection using GPS wearable data. This study will potentially drive interest in conducting further studies on different populations and in various sporting fields using similar methods to better understand the potential impact of the SARS-CoV-2 virus on the exercise activity of athletes.

## Methods

### Study design and participants

The study is an observational study on recreational runners and cyclists predominantly from South Africa (two international participants were included). Participants were recruited using convenient, non-randomised sampling, via emailing running and cycling clubs in South Africa, and advertising the study on social media and at the Sports Science Institute of South Africa. The advertisement included a basic description of the study and a link to the study’s website. The website page contained links to the study’s participation information sheet containing instructions on how to download their GPS data, the informed consent form, and a study questionnaire via Google forms.

Inclusion criteria were runners and cyclists over the age of 18 that reportedly tested positive for COVID-19, had attempted to return to exercise post infection, and used a GPS wearable device that measures heart rate. A control group that had not been infected with COVID-19 but had an interruption in training of two weeks or more was also included in the study. Training interruption was defined as a complete cessation of running or cycling training, not associated with COVID-19 infection.

All participants provided informed consent via the study’s online Google forms questionnaire. The study received ethics approval from the Faculty of Health Sciences Human Research Ethics Committee at the University of Cape Town (Ref. No. 409/2021). This study complies with the latest version of the Declaration of Helsinki (2013), as well as the Department of Health: Ethics in Health Research: Principles Structures and Processes (2004).

This study was a “proof-of-concept study”. Thus, a sample size calculation was not performed.

### Questionnaire

A questionnaire on Google forms was provided for the participants to complete. Demographic, health, and training self-reported data were obtained. For the 42 persons who reported having tested positive for COVID-19, additional data were captured, including the duration of their COVID-19, COVID-19 symptoms, and COVID-19 severity. For the control group, the reason for their training interruption and training interruption duration were also obtained.

### GPS data

Participants were required to download their GPS wearable device data by following the instructions provided in the participation information sheet. Thereafter, participants sent their training data to the researcher to be analysed. Participants were required to send GPS data from four weeks pre-training interruption to four weeks post return to training.

Raw data were sorted and filtered on Microsoft Excel (version 2110) to include the following variables: average and peak heart rate during training per week (bpm), average and maximum speed per week (min/km), total time training per week (min), total distance per week (km), with variables being averaged for each week. Change in training activity was measured by comparing the above training variables pre and post-training interruption. Relative exercise intensity was determined by calculating age-corrected maximum heart rate using the HUNT equation (211–0.64*age)^[7]^ and dividing the average heart rate for each week by the maximum heart rate.

### Statistical analysis

The program IBM SPSS Statistics 27 and GraphPad Prism 9 were used to conduct statistical analyses. Data were expressed as number and percentage for categorical variables and mean and standard deviation for continuous variables. Pearson Chi-squared tests and independent t-tests were used to determine any differences in categorical and continuous variables between the two groups. Repeated measures analysis of variance (ANOVA) was run to determine within-group and between-group differences in the GPS data of participants. Correlations were determined using Spearman’s rank correlation tests. Statistical significance was set at a P-value of <0.05.

## Results

A total of 61 participants provided consent to participate in the study and completed the questionnaires, with 42 individuals being in the COVID-19 group, and 19 in the control group. Of those that completed the questionnaire, 38 participants provided their GPS data (COVID-19: N=27; Controls: N= 11). The data of two control participants were removed prior to analysis due to training data not meeting inclusion criteria, reducing the GPS control sample to nine.

### Questionnaire

#### COVID-19 and control group characteristics

No statistically significant differences were found in baseline characteristics of participants (p>0.05) (Table 1). Most participants in both groups were above the age of 40 (~70%), were male (~74%), had a normal BMI (~67%), and were runners (~48%). Most participants used Garmin wearable devices (without a chest strap to measure heart rate, ~59%). The most frequent training interruption length was between 2–4 weeks for the COVID-19 group (45%), and 1–3 months for the control group (32%) (p=0.054). The most common cause of training interruption for the control group was COVID-19 lockdown restrictions.

#### COVID-19 group characteristics

Table 2 shows health and COVID-19 related characteristics of the COVID-19 group. Most participants had no comorbidities (69%) and were unvaccinated before and after being infected with COVID-19 (57%). The most frequent acute COVID-19 symptoms reported were headaches (79%), body aches (74%), and fatigue (74%). Symptom duration was most frequently between 0–2 weeks (60%). COVID-19 severity was mild to moderate for most participants (91%) with only one participant being hospitalised. The type of treatment reported varied, with most participants using over-the-counter medication such as paracetamol (56%). About 58% of participants said that they followed guidelines for return to training from their doctors. However, only 16% of participants reported being medically screened before returning to exercise. The most frequent symptoms reported when returning to training were increased heart rate (58%), and fatigue (53%).

Positive correlations were found between COVID-19 severity and number of symptoms (r=0.50 [95% CI= 0.21–0.70]; p=0.001), symptom duration (r=0.55 [95% CI=0.27–0.74]; p<0.001), and training interruption time (r=0.52 [95% CI= 0.24–0.72]; p<0.001). There were also correlations between number of symptoms and symptom duration (r=0.42 [95% CI=0.12–0.65]; p=0.006), and interruption time (r=0.49 [95% CI=0.20–0.70]; p=0.001).

### GPS data

#### Training interruption time

There was a statistically significant difference in the training interruption time between the COVID-19 group and the control group with GPS data. The control group had a longer training interruption time than the COVID-19 participants (33 ± 11 days vs 20 ± 13 days, p=0.014) As mentioned previously, the most common reason for a training interruption for the control group was lockdown restrictions, with other reasons for training interruptions including injury and illnesses other than COVID-19. There were no other differences in baseline characteristics such as age, sex, and BMI.

#### Peak heart rate, average heart rate, and relative exercise intensity

Decreases in peak and average heart rate, and relative exercise intensity were observed in both groups, one-week post return to training, compared to one-week pre-training interruption. (Mean peak heart rate: COVID-19:171 beats per minute (bpm) to 158 bpm; Control:178 bpm to 161 bpm. Mean average heart rate: COVID-19:147 bpm to 140 bpm; Control:146 bpm to 138 bpm. Mean relative exercise intensity: COVID-19:80% to 76%; Control: 82%–77%). These decreases were statistically significant in both groups for peak heart rate (p=0.017), but not statistically significant for average heart rate and relative exercise intensity (p=0.095; p=0.091).

**Fig. 1:**
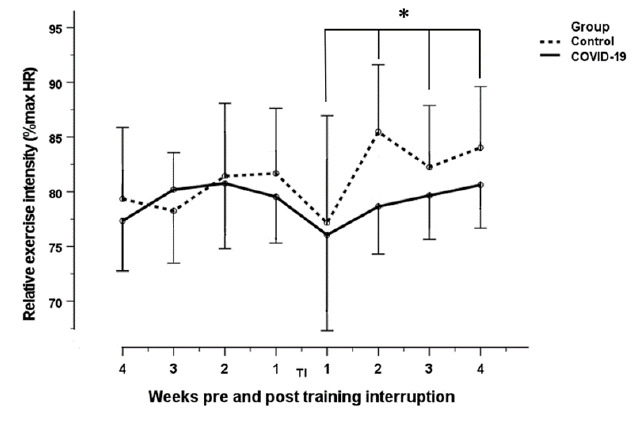
Change in relative exercise intensity of COVID-19 and control groups four weeks pre interruption to four weeks after return to training post interruption; *statistical significant differences in relative exercise intensity between one week post return to training, and two, three and four weeks post return to training (p=0.03); TI: training interruption period; error bars 95% confidence interval.

Following the above decreases at one week post return to training, increases in peak and average heart rate, and relative exercise intensity (Figure 1) were observed in both groups from the second to the fourth week post return to training (Mean peak heart rate: COVID-19: 158 bpm to 172 bpm; Control: 160 bpm to 177 bpm. Mean average heart rate: COVID-19: 140 bpm to 149 bpm; Control: 138 bpm to 150 bpm. Relative exercise intensity: COVID-19: 76% to 80%; Control: 77% to 84%) These increases were statistically significant in both groups for all three variables (p<0.04), and variables increased close to their original values pre-training interruption. No between-group differences were found for peak heart rate, average heart rate and relative exercise intensity (p=0.182; p=0.360; p=0.338).

#### Maximum and average speed

Maximum and average speed were analysed separately for runners and cyclists due to the difference in the nature of training modality (cyclists’ speed is naturally faster than runners’ speed). Cyclists’ maximum and average speeds were omitted from analysis due to a very small sample size as a result of missing data values (n=6).

**Fig. 2:**
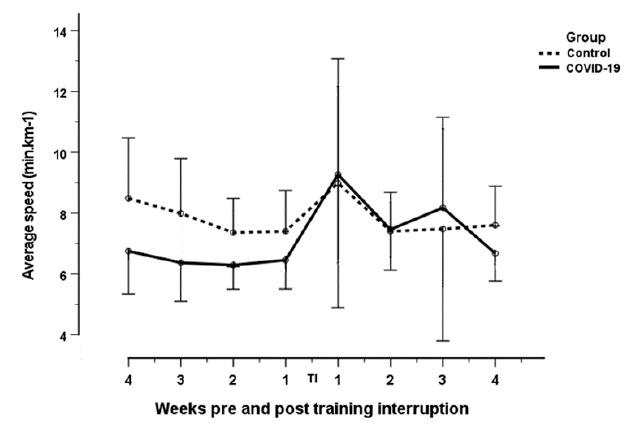
Change in average speed of COVID-19 and control groups four weeks pre-interruption to four weeks after return to training post interruption. TI: Training interruption period; error bars 95% confidence interval.

A non-statistically significant decrease in maximum and average speed was observed (increase in min/km) for runners in both groups, at one week post return to training, compared to one week pre-training interruption (Mean maximum speed: COVID-19: 4.36 min.km^−1^ to 5.99 min.km^−1^; Control: 5.41 min.km^−1^ to 5.98 min.km^−1^; p=0.07). Mean average speed:COVID^−^19: 6.45 min.km^−1^ to 9.26 min.km^−1^; Control: 7.40 min.km^−1^ to 8.98 min.km^−1^; p=0.066). This was followed by a non-statistically significant increase in maximum and average speeds at Weeks two to four post return to training. The COVID-19 group had a faster average speed than the control group pre-training interruption (Figure 2.). However, no statistically significant group effect was found at any point in training (p=0.132).

#### Training time and distance

**Fig. 3:**
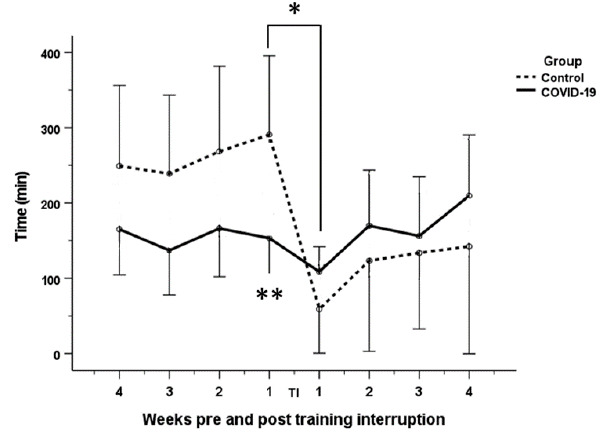
Change in time trained of COVID-19 and control groups four weeks pre-interruption to four weeks after return to training post interruption; *statistical significant difference in time trained for the control group, between one week pre-training interruption and one week post return to training (p<0.001);**statistical significant difference in time trained between the two groups at one week pre-training interruption (p=0.002) TI: Training interruption period; error bars 95% confidence interval.

**Fig. 4:**
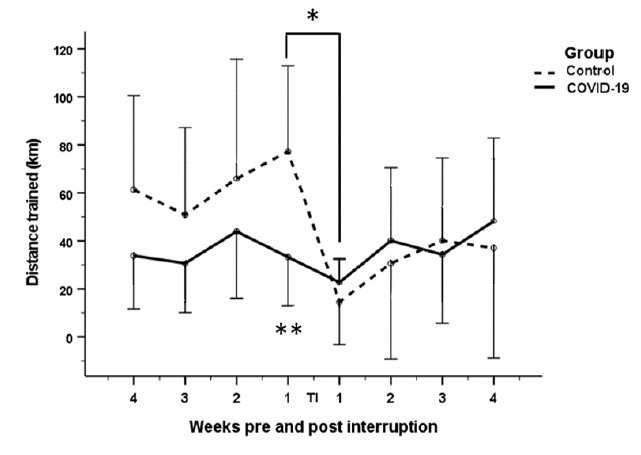
Change in distance trained of COVID-19 and control groups four weeks pre-interruption to four weeks after return to training post interruption; *statistical significant difference in distance trained for the control group, between one week pre-training interruption and one week post return to training (p=0.002);**statistical significant difference in distance trained between the two groups at one week pre-training interruption (p=0.003) TI: Training interruption period; error bars 95% confidence interval.

A statistically significant decrease in training time (p<0.001) and distance (p=0.002) was observed in the control group, one week post return to training compared to one week pre-training interruption (Time trained: 291 min to 59 min; Distance: 77 km to 23 km). In contrast, a non-statistically significant decrease in time and distance was observed in the COVID-19 group, at one week post return to training compared to one week pre-training interruption (Time: 153 min to 109 min; Distance: 33 km to 14 km). Additionally, the distance and time trained at one week pre-training interruption were statistically significantly different between the two groups (p=0.002; p=0.003) (Figures 3 and 4).

The decreases in both groups were followed by increases in time and distance trained from Week two to four post return to training. However, these increases were not statistically significant for either group. For the COVID-19 group, the participants appeared to recover their pre-interruption training time and distance levels. However, the control group’s training time and distance remained lower than their pre-training interruption values (Figures 3 and 4).

## Discussion

### Questionnaire

#### COVID-19 clinical presentations

The findings of this study show that the COVID-19 clinical presentation in recreational runners and cyclists was mild to moderate (90%) with fatigue, body ache and headache being the most common symptoms, and 0–2 weeks being the most frequent symptom duration. This clinical presentation correlates with findings in previous studies on athletes post COVID-19 infection. Studies by Schwellnus et al.^[10]^ and Hull et al.^[11]^ both observed headache and fatigue as one of the most prevalent COVID-19 symptoms in athletes, with athletes having predominantly mild illness severity.^[10,11]^ A possible reason for the milder illness severity found in athletes is that regular exercise of moderate intensity can potentially improve one’s immune response to infection, decrease inflammation, and reduce the risk of metabolic conditions, such as diabetes and obesity, which are considered risk factors for severe COVID-19.^[12]^ The correlations with COVID-19 severity found in this study also suggest that the more severe the illness, the longer the training interruption time, the greater the number of symptoms, and the longer the symptom duration.

### GPS data

In the first week of the return to training, decreases in peak and average heart rate, relative exercise intensity, maximum and average speed, time, and distance were observed for both the COVID-19 and control groups, compared to one week pre-training interruption. Because these decreases were observed in both the COVID-19 and control groups, this suggests that there was no specific ‘COVID-19’ effect on training activity post infection in this group of athletes. The above decreases were then followed by increases in all training variables at Weeks two to four post return to training.

A possible reason for the decreases in the measured training variables at Week one post return to training, could be a conscious choice of the athletes to start training at a lower volume and intensity compared to before the COVID-19 infection and training interruption. This hypothesis is further strengthened by the ability of athletes to increase their training values back to pre-training interruption values, suggesting a maintained exercise ability. Furthermore, 58% of participants in the COVID-19 group said that they followed the guidelines for return to training from their doctors, which could also be a reason for their gradual return to training. Based on the results, it could be suggested that following a gradual return to training post COVID-19 infection is a safe and beneficial way to approach returning to training, as participants were able to return to their original levels of training by four weeks post training interruption.

In contrast, the control group did not return to their original values for time and distance trained, with values being lower than pre-training interruption values. This could possibly be due to the control group having a longer time off than the COVID-19 group (33 ± 11 days vs 20 ± 13 days, p=0.014), thus making it more difficult to return to original values pre-training interruption. This could potentially be due to a detraining effect. Detraining is the partial or complete reversal of physiological adaptations induced from training, due to a reduction or cessation of training stimuli.^[13]^ However, the peak and average heart rates and relative exercise intensity after the training interruption remained similar to the pre-training interruption values. One would expect an increase in heart rate if detraining occurred. Therefore, it is more likely that the control group was less motivated to return to their original levels of exercise due to the prolonged time off training, as opposed to a detraining effect.

In conclusion, the data above suggest that COVID-19 infection is associated with an interruption in training in recreational runners and cyclists. However, COVID-19 did not have a more serious effect on return to training compared to other forms of training interruption.

### Study limitations

This study has several limitations that may influence the validity of the results. Many of the changes in training variables over time were not statistically significant, with the confidence intervals of training values being wide. This is most likely due to the small sample sizes. Furthermore, the study did not include any objectively physiologically measured data, such as data from cardiopulmonary exercise testing. Researchers were also limited by the data provided by the participants, which contained a limited number of training variables. Having variables such as resting heart rate and time trial data would have been beneficial. Additionally, the training load pre-training interruption and the training interruption time of the COVID-19 and control groups were not matched, with the control group having a larger time and distance trained pre-training interruption and a longer training interruption time. Additionally, GPS wearable heart rate measurements are known to often be inaccurate when the optical wrist-based pickup is used rather than a chest strap.^[14]^ Furthermore, equations used to calculate age-corrected maximum heart rate could result in overestimations or underestimations of readings.^[15]^

Other limitations include the small sample size of participants, which further decreased during the analysis due to missing data values. In addition, the control group’s sample size is much smaller than the COVID-19 group, thus decreasing the comparator group effect. Despite its limitations, this study shows the value of using GPS wearable training data to determine the effect of COVID-19 on the training activity of athletes post infection. Future studies could improve on the current study by recruiting a larger number of participants, including resting heart rate and time trial data and matching the training loads and interruption times of cases and controls.

## Conclusion

To our knowledge, this is the first study investigating the effect of COVID-19 on the training activity of recreational athletes using GPS data. Most participants had mild to moderate COVID-19, with associations found between COVID-19 severity and number of symptoms, symptom duration, and training interruption time. COVID-19 was also associated with a self-reported training interruption time of two-four weeks. At one week post training interruption decreases in peak and average heart rate, relative exercise intensity, maximum and average speed, time and distance trained were observed for both the COVID-19 and control groups. This was followed by an increase in these variables between two to four weeks’ post return to training in both groups. The decreases in training variables were observed for both groups, thus eliminating the possibility of a specific ‘COVID-19 effect’ on training activity post infection. A possible reason for the pattern of changes observed in training variables post COVID-19 could be participants deliberately returning to exercise at lower volumes and intensities in order to return to training safely. The study demonstrates the value of using GPS wearable device data to evaluate athletes’ training activity post training interruptions. However, due to the many limitations of the study, the results should be taken with caution, with more research being required to further expand on the study's results.

## Figures and Tables

**Table 1:** Demographic and training characteristics of the COVID-19 and control group

Characteristics	COVID-19 n=42	Control n=19	P value
**Age (years)**			0.806
19–29	3 (7)	1 (5)	
30–40	11 (26)	4 (21)	
40–50	17 (41)	6 (32)	
50–60	10 (24)	7 (37)	
>60	1 (2)	1 (5)	

**Sex**			0.114
Male	27 (64)	16 (84)	
Female	15 (36)	3 (16)	

**BMI (kg/m** ^**2**^ **)**			0.605
Underweight	1 (2)	0 (0)	
Normal weight	28 (67)	13 (68)	
Overweight	10 (24)	3 (16)	
Obese	3 (7)	3 (16)	

**Sport**			0.985
Runner	20 (48)	9 (47)	
Cyclist	5 (12)	2 (11)	
Runner and Cyclist	17 (41)	8 (42)	

**Chest strap**			0.539
Yes	19 (45)	7 (20)	
No	23 (55)	12 (63)	

**Training interruption time (weeks)**			0.054
0–2 weeks	10 (24)	1 (5)	
2–4 weeks	19 (45)	5 (26)	
1–3 months	13 (31)	8 (32)	
>3months	0 (0)	5 (26)	

**Reasons for training interruption of control group**			
Lockdown restrictions	14 (74)		
Illness (excluding COVID-19)	1 (5)		
Injury	1 (5)		
Other	3 (16)		

Data are shown as the number of participants and column percentage (%). P values from Pearson Chi-squared test. Underweight, <18.5kg/m^2^; normal weight, 18.5–29.9kg/m^2^; Overweight, 25–29.9kg/m^2^; Obese, >30kg/m^2^.

**Table 2:** Comorbidities, COVID-19 presentation, and treatment in the COVID-19 group (N=42)

		N (%)
**Comorbidities** *	Diabetes	1 (2)
	High Blood pressure	3 (7)
	Cholesterol	4 (10)
	Obesity	1 (2)
	Asthma	8 (19)
	Autoimmune disease	0 (0)
	None	29 (69)

**COVID-19 presentation**	**COVID-19 Symptoms** *	
	Cough	25 (58)
	Fever	23 (54)
	Sore throat	21 (49)
	Fatigue	32 (74)
	Body aches	31 (74)
	Loss of taste or smell	21 (49)
	Headache	34 (79)
	Diarrhoea	8 (19)
	Difficulty breathing	12 (28)
	Chest pain	12 (28)
	Other	4 (9)
	None	1 (2)

	**Number of symptoms**	5 ± 2

	**Symptom duration**	
	0–2 weeks	25 (60)
	2–4 weeks	12 (29)
	1–3 months	2 (5)
	>3 months	3 (7)

	**COVID-19 Severity**	
	Asymptomatic	2 (5)
	Mild	20 (48)
	Moderate	18 (43)
	Severe	2 (5)
	Critical	0 (0)

	**Symptoms when returning to training** *	
	Cough	3 (7)
	Fatigue	23 (54)
	Shortness of Breath	12 (28)
	Increased Heart Rate	25 (58)
	Other	3 (7)
	None	6 (14)

**Treatment and protective measures**	**Hospitalisation**	
	Yes	1 (2)
	No	41 (98)

	**Treatment** *	
	Corticosteroids	8 (18)
	Antibiotics	7 (16)
	Oxygen	2 (5)
	Over the counter medication	24 (56)
	Ivermectin	4 (10)
	Vitamins/Supplements	15 (35)
	None	6 (14)

	**Fully vaccinated**	
	Pre COVID-19	3 (7)
	Post COVID-19	15 (36)
	Not vaccinated	24 (57)

	**Guidelines followed before returning to training**	
	Yes, guidelines from medical practitioner	23 (58)
	Yes, guidelines from scientific journal articles	3 (7)
	Yes, guidelines from internet	3 (7)
	No	13 (31)

	**Medical screening before return to training**	
	Yes	7 (17)
	No	35 (83)

Data are shown as the number of participants and percentage (%) or as mean ± SD.

* participants can fall into multiple categories.

COVID-19 severity: Asymptomatic, no symptoms but tested positive for COVID-19; Mild, had flu-like symptoms excluding shortness of breath at rest or during exertion; Moderate, flu-like symptoms and/or shortness of breath, may or may not be hospitalized; Severe, requires hospitalization and oxygen administration; Critical, requires hospitalization and ventilation, multi-organ involvement may be present.^[8,9]^
